# Comparative transcriptomics of the Djungarian hamster hypothalamus during short photoperiod acclimation and spontaneous torpor

**DOI:** 10.1002/2211-5463.13350

**Published:** 2021-12-20

**Authors:** Elena Haugg, Janus Borner, Victoria Diedrich, Annika Herwig

**Affiliations:** ^1^ Institute of Neurobiology Ulm University Germany; ^2^ Institute of Evolutionary Ecology and Conservation Genomics Ulm University Germany; ^3^ Sackler Institute for Comparative Genomics American Museum of Natural History New York NY USA

**Keywords:** RNA‐Seq, gene expression, metabolism, hypothermia, Siberian hamster, *Phodopus sungorus*

## Abstract

The energy‐saving strategy of Djungarian hamsters (*Phodopus sungorus,* Cricetidae) to overcome harsh environmental conditions comprises of behavioral, morphological, and physiological adjustments, including spontaneous daily torpor, a metabolic downstate. These acclimatizations are triggered by short photoperiod and orchestrated by the hypothalamus. Key mechanisms of long‐term photoperiodic acclimatizations have partly been described, but specific mechanisms that acutely control torpor remain incomplete. Here, we performed comparative transcriptome analysis on hypothalamus of normometabolic hamsters in their summer‐ and winter‐like state to enable us to identify changes in gene expression during photoperiodic acclimations. Comparing nontorpid and torpid hamsters may also be able to pin down mechanisms relevant for torpor control. A *de novo* assembled transcriptome of the hypothalamus was generated from hamsters acclimated to long photoperiod or to short photoperiod. The hamsters were sampled either during long photoperiod normothermia, short photoperiod normothermia, or short photoperiod‐induced spontaneous torpor with a body temperature of 24.6 ± 1.0 °C, or. The mRNA‐seq analysis revealed that 32 and 759 genes were differentially expressed during photoperiod or torpor, respectively. Biological processes were not enriched during photoperiodic acclimatization but were during torpor, where transcriptional and metabolic processes were reinforced. Most extremely regulated genes (those genes with |log2(FC)| > 2.0 and padj < 0.05 of a pairwise group comparison) underpinned the role of known key players in photoperiodic comparison, but these genes exhibit adaptive and protective adjustments during torpor. Targeted analyses of genes from potentially involved hypothalamic systems identified gene regulation of previously described torpor‐relevant systems and a potential involvement of glucose transport.

## Graphical abstract

Djungarian hamsters (*Phodopus sungorus*) use morphological and physiological changes to overcome extreme environmental conditions. These acclimatizations include spontaneous daily torpor. To uncover the mechanisms behind torpor, samples were collected from nontorpid and torpid hamsters during summer‐ or winter‐like photoperiod. The pipeline comprised a *de novo* transcriptome assembly, a mapping against *Mus musculus*, and several analyses to identify the most relevant data. Analysis of the hypothalamus transcriptome confirms known and suggests new mechanisms that might orchestrate seasonal acclimation and metabolic downstates in photoperiodic rodents.

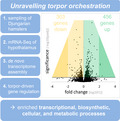

**Abbreviations**
HT: hypothermia
log2(FC): logarithm of fold change to the base 2
LP: summer‐like long photoperiod
NT: normothermia
padj: adjusted *P*‐value
SP: winter‐like short photoperiod
Tb: core body temperature
ZT: *Zeitgeber‐time*

Djungarian hamsters (*Phodopus sungorus,* Cricetidae) are photoperiodic mammals [1, 2]. In summer or summer‐like long photoperiod (LP; e.g., 16:8 light:darkness), they have a high body mass, body temperature, and activity level. They are reproductively active and camouflaged to their environment by a light brown fur. As soon as photophase decreases below 13 h of light per day during autumn in the field [3], or after a transfer to winter‐like short photoperiod in captivity (SP; e.g., 8:16 light:darkness), the hamsters reduce body temperature, activity during scotophase, food intake, and body mass. They recede their gonads and stop to reproduce. They grow a dense white winter fur which improves insulation capacity [4, 5, 6]. The manifestation of these acclimation parameters is highly individual and therefore variable within a cohort [6].

After about ten weeks of short photoperiod exposure, the individual acclimation reaches a certain threshold. 75% of hamsters start to express spontaneous daily torpor, even at 20 °C ambient temperature with food and water *ad libitum* [6]. Torpor is characterized by a precisely regulated natural reduction of metabolism resulting in a body temperature decrease below normothermic values, so‐called hypothermia [7], for several hours during photophase [8]. On average, torpor is expressed on two days of a representative week. However, torpor behavior is highly individual [6].

Morphological, behavioral, and physiological acclimatizations to environmental cues such as day length are elements of a complex energy‐saving strategy that is orchestrated within the hypothalamus by the photoperiodic neuroendocrine system including the suprachiasmatic nucleus, the endogenous mammalian photic, and circadian pacemaker [9, 10, 11]. The underlying regulatory mechanisms of photoperiod‐controlled adjustments of body mass and reproduction in *Phodopus* have been extensively studied and were recently reviewed [12]. They involve gene expression changes in the thyroid system via the *Pars tuberalis* of the pituitary gland, in energy metabolism, mediated by glucose‐sensitive tanycytes lining the third ventricle, and in the signaling of hypothalamic neurons sensitive, for example, *POMC, NPY, AGRP,* and *CARTPT,* which drive orexigenic and anorexigenic physiological responses [13, 14]. Since the expression of torpor is dependent on signaling of various hormonal systems changing with photoperiodic as well as nutritional state and circadian rhythm, it can be assumed that the hypothalamus is also involved in its proximate control. Although several hypothalamic systems are likely to regulate torpor, gene expression studies using either whole transcriptome approaches or targeted gene expression analyses could not yet nail down specific mechanisms [15, 16].

In a previous NGS approach, we tried to identify signaling mechanisms within the hamsters’ hypothalamus during torpor entrance [17]. However, the data revealed general molecular adjustments likely maintaining functional integrity, rather than signaling mechanisms. It is unclear which thresholds are applicable to pin down physiologically relevant genes. In general, transcriptome analyses in a nonmodel organism such as the Djungarian hamster are challenging given the lack of both an annotated reference genome and standardized, adequate bioinformatical as well as statistical pipelines [17, 18]. In the present study, we used the hamsters as physiology‐based model organism for seasonal acclimation and torpor research, in which some of the underlying hypothalamic signaling mechanisms have already been disentangled. We sequenced hypothalami of summer‐like long photoperiod‐ and winter‐like short photoperiod‐acclimated hamsters in their normothermic state, as well as of short photoperiod‐acclimated hamsters during torpor. With this data set, we tried to define parameters allowing us to confirm already known gene expression changes in a photoperiodic context. We assumed that the applied methods would also identify genes that are likely to have biological relevance in hypothalamic torpor control. Despite enrichment analyses and most extremely regulated genes, we specifically screened for indicator genes that were preselected from hypothalamic systems potentially involved in torpor control, such as the circadian system, thyroid system, growth axis, and metabolic energy balance. [12]

## Materials and methods

### Breeding and housing

Twelve Djungarian hamsters *(Phodopus sungorus)* were bred in 2019 according to an outbred crossing scheme in the indoor breeding colony at the Institute of Neurobiology (Ulm University, Germany) in accordance with the local ethics committee (35/9185.46‐3). Ambient temperature was maintained at 20 ± 1 °C. Artificial light (150 lux) was provided 16 h per day in summer‐like long photoperiod (LP). Additional constant red light (< 5 lux) enabled animal handling during the scotophase. Tap water and food (Altromin hamster breeding diet 7014, Lage, Germany) were provided *ad libitum*, supplemented by cucumber, oat flakes, and sunflower seeds once a week. Adult hamsters were single housed in Makrolon Type III cages (26.5 × 42.5 × 18.0 cm) with wooden bedding and tissue as nesting material.

### Radiotelemetry

To assess spontaneous torpor expression, the core body temperature (Tb) was monitored in real time in a resolution of 3 min using a radiotelemetry system with DataQuest™ artbronze software (DSI—Data Sciences International, Harvard Bioscience Inc., St. Paul, MN, USA). A receiver board (RPC1) was positioned under each individual home cage. A transmitter (model TA‐11TA‐F10, silicone‐coated, 1.1 cc volume, 1.6 g weight, 0.15 °C accuracy) was implanted intraperitoneally under isoflurane anesthesia (2.5% and 1 mL·min^−1^ for induction, 0.75 to 2.0% and 0.4 mL·min^−1^ for maintenance) and carprofen analgesia (5 mg·kg^−1^ i.p. Rimadyl®, Zoetis Deutschland GmbH, Berlin). Recovery from surgery was supported by additional oat flakes, sunflower seeds, cucumber, and nesting material. Body mass, fur care, posture, and behavior were monitored daily for about seven days until recovery. Experimental and surgical procedures were approved by the Regierungspräsidium Tübingen, Germany (1411). Radiotelemetry data of hamsters were analyzed previously [6].

### Key data of hamsters

Four of the twelve hamsters remained in LP, had an initial body mass of 33 ± 4 g, and were not implanted with DSI transmitters. They were sacrificed with a body mass of 34 ± 4 g at an age of 30 ± 1 weeks. Eight of the twelve hamsters were transferred to winter‐like short photoperiod (SP) with 8 h of light per day at an age of 17 ± 3 weeks, were implanted with DSI transmitters at an age of 29 ± 2 weeks, and sacrificed two to eight weeks later at an age of 33 ± 4 weeks. During the 16 ± 2 weeks of SP acclimation, they reduced their body mass by 21 ± 13% to 27 ± 2 g. Background information on each hamster is provided (Table S1).

### Sampling scheme

**Fig. 1:**
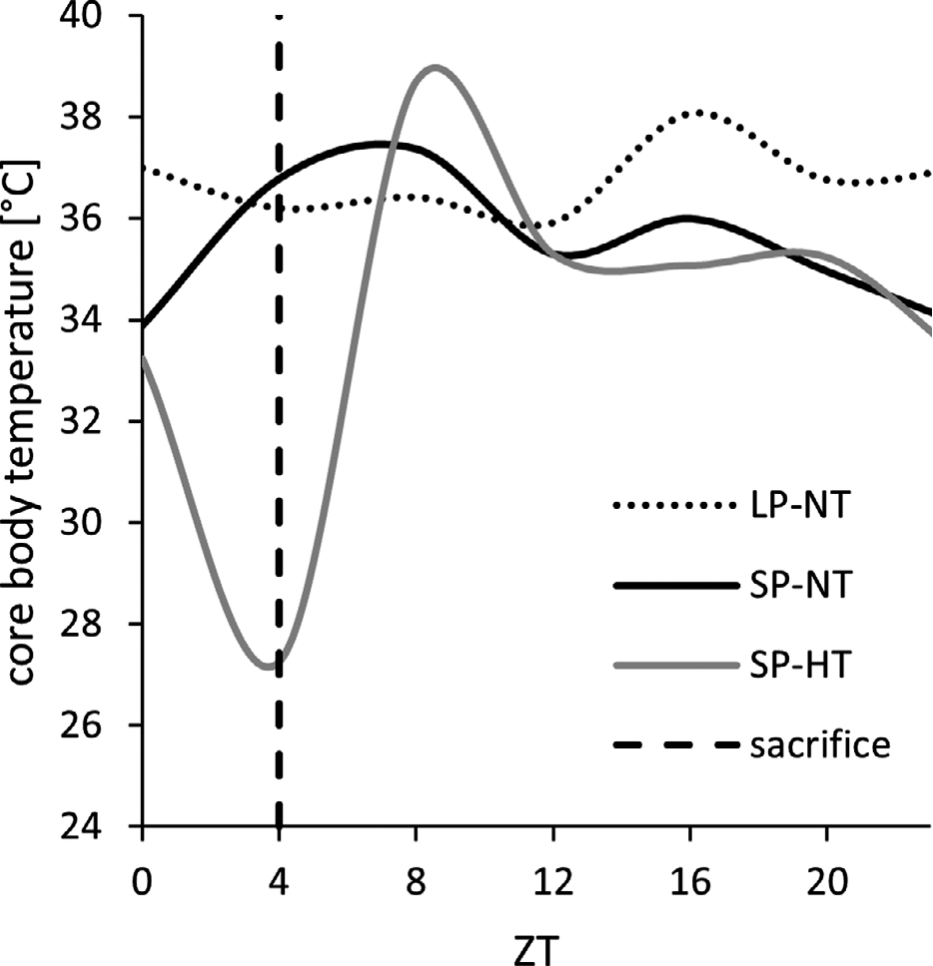
Exemplary core body temperature patterns. Raw data measured in intervals of 3 min were processed to mean body temperature per hour and displayed in intervals of 4 h. The hamsters of this study were sacrificed around ZT04 (vertical line). Individual core body temperature patterns of SP‐acclimated hamsters are attached in Fig. S1. LP‐acclimated hamsters of this study were not implanted. Plotted data were derived from another studies’ hamster which was monitored in LP as well as SP with food and water *ad libitum* at 20 °C ambient temperature [6].

All twelve hamsters were sacrificed 4 h after the beginning of the photophase at approximately ZT04 (Fig. 1) in three sampling groups: either SP‐hypothermic at the nadir of torpor (SP‐HT, *n* = 4, three males, one female, Tb = 24.6 ± 1.0 °C), SP‐normothermic (SP‐NT, *n* = 4, two males, two females, Tb = 35.3 ± 0.3 °C), or LP‐normothermic (LP‐NT, *n* = 4, two males, two females, not implanted). Before sampling, all SP‐acclimated hamsters had expressed at least one torpor bout (Table S1). Spontaneous daily torpor was defined as a core body temperature below 32 °C for at least 30 min [16, 19, 20]. The body temperature patterns of the last day and the day before are provided (Fig. S1). All hamsters were sacrificed with carbon dioxide. The circulatory system was perfused transcardially with 0.1 M sterile PBS (pH = 7.42). To reduce the number of laboratory animals, the hamsters were dedicated to several studies on other organs taken after decapitation (unpublished data).

### Dissection of hypothalamus

The brain was quickly removed, shock frozen on dry ice, and stored at −80 °C. For further processing, the brains were trimmed in a cryochamber of −17 °C with a razor blade. The hypothalamus was clearly visible as a central elevation on the ventral side of the brain expanding from Bregma 0.38 mm to −2.80 mm (according to the mouse brain atlas [21]). The hypothalamus was first trimmed to a tissue cuboid cutting from ventral to dorsal. To remove the thalamus and the cortex, brains were cut from anterior to posterior directly on the anterior part of the anterior commissure, visible as most ventral bilateral white matter. Edges of the tissue cuboid were removed cutting from ventral to dorsal to resemble the cylindric shape of the hypothalamus.

### Purification of total RNA, isolation, and sequencing of mRNA

Hypothalamic tissue was homogenized in 100 µL buffer RLT with beta‐ME using prefilled 2‐mL beadbug tubes containing Zirconium beads (Biozym Scientific GmbH, Oldendorff, Germany) on a microtube homogenizer (D1030‐E, Benchmark Scientific Inc., Sayreville, NJ, USA). Purification of total RNA was performed using the RNeasy Midi Kit (Qiagen GmbH, Hilden, Germany). The concentration of total RNA ranged between 15 and 86 ng·µL^−1^ (56 ± 23 ng·µL^−1^; NanoDrop 2000 spectrophotometer, Thermo Fisher Scientific GmbH, Dreieich, Germany). For further processing, the samples were sent to StarSEQ GmbH, Mainz, Germany. The RNA integrity number (RIN) varied between 7.8 and 9.5 (8.5 ± 0.5). The rRNA ratio [28 s/18 s] varied between 1.4 and 1.8 (1.7 ± 0.1). The mRNA was isolated and the library prepared (NEBNext® Ultra™ II directional, dual index RNA). RNA‐Seq was performed in 2020 on an Illumina NextSeq 500 (1 lane, 50 mio paired end reads, 2 × 25 mio reads, 2 × 150 nt, 7.5 gb).

### Bioinformatics

Bioinformatics were performed on the bwForCluster NEMO of the Baden‐Württemberg High Performance Computing (bwHPC) project. After quality control of each fastq file using FastQC 0.11.9 [22], on average, 32,717,097 read pairs per sample (Table S2) were processed in several steps. Data of each sample were prepared with trim‐galore 0.6.6 [23]. In total, 392,605,160 read pairs from all samples were pooled to generate a single *de novo* transcriptome assembly containing 868,657 transcripts using Trinity 2.8.5 [24, 25]. The transcripts were mapped against the reference proteome of *Mus musculus* (GRCm39, Annotation Release 109) using blastx 2.5.0+ with an e‐value < 1E‐5 [26, 27]. In total, 171,923 transcripts of the hamster assembly were successfully mapped against the reference. A reduced assembly containing only transcripts with a hit against a mouse protein was generated using ‘extract_hits_from_fasta.rb’. A transcript‐to‐GeneID dictionary was generated based on the best hit to the mouse proteome by using ‘hits_to_genemap.rb’. The reads from each sample were then mapped back to the reduced assembly using bowtie 1.3.0 [28]. The non‐normalized differential gene expression was calculated for each sample using rsem 1.3.1 [29].

### Data depositories and supplied scripts

The raw Illumina data have been deposited at the NCBI Sequence Read Archive under SRA Study accession number SRP326941 within the Bioproject PRJNA743775. Both, the original and the processed *de novo* assembly and the non‐normalized gene expression have been deposited in NCBI's Gene Expression Omnibus [30]. These data are accessible through GEO Series accession number GSE179663 (https://www.ncbi.nlm.nih.gov/geo/query/acc.cgi?acc=GSE179663). An overview of accession numbers within SRA and GEO is attached as Table S2. The data processing pipeline from raw Illumina data to non‐normalized gene expression are attached as Doc S2. Both ruby‐scripts were uploaded to SourceForge (https://sourceforge.net/projects/prepare‐transcript‐to‐gene‐map/). The R‐script with R‐session info is attached as Doc S3.

### Statistics

Statistics and principal component analysis were performed in RStudio 3.5.2 [31, 32] using DESeq2 [33, 34, 35]. Data were normalized across all samples of a pairwise group comparison to level methodical bias. In this study, two pairwise group comparisons were conducted: NT: SP vs LP to reveal photoperiod‐driven differential gene expression of normothermic Djungarian hamsters, and SP: HT vs NT to detect torpor‐driven differential gene expression of SP‐acclimated Djungarian hamsters. Processing and graphical representation of normalized data was performed with Microsoft Excel (Office 365, 2016).

### Data interpretation

Data analysis was based on the numerical GeneID. One GeneID includes all isoforms, precursors, and preproproteins. Per gene present with at least ten counts in a pairwise group comparison, the averaged count per group was compared with the averaged count of the other group, resulting in the gene’s fold change, provided as log2(FC), and the significance of this fold change (adjusted *P*‐value, padj). A negative fold change indicates a downregulated gene, a positive fold change an upregulated gene. A log2(FC) of −1 indicates half the expression in the first‐named compared with the second‐named group of a pairwise comparison, while a log2(FC) of 1 indicates a doubled expression, a log2(FC) of 2 a quadrupled expression, etc.

### Gene expression profiling

**Fig. 2:**
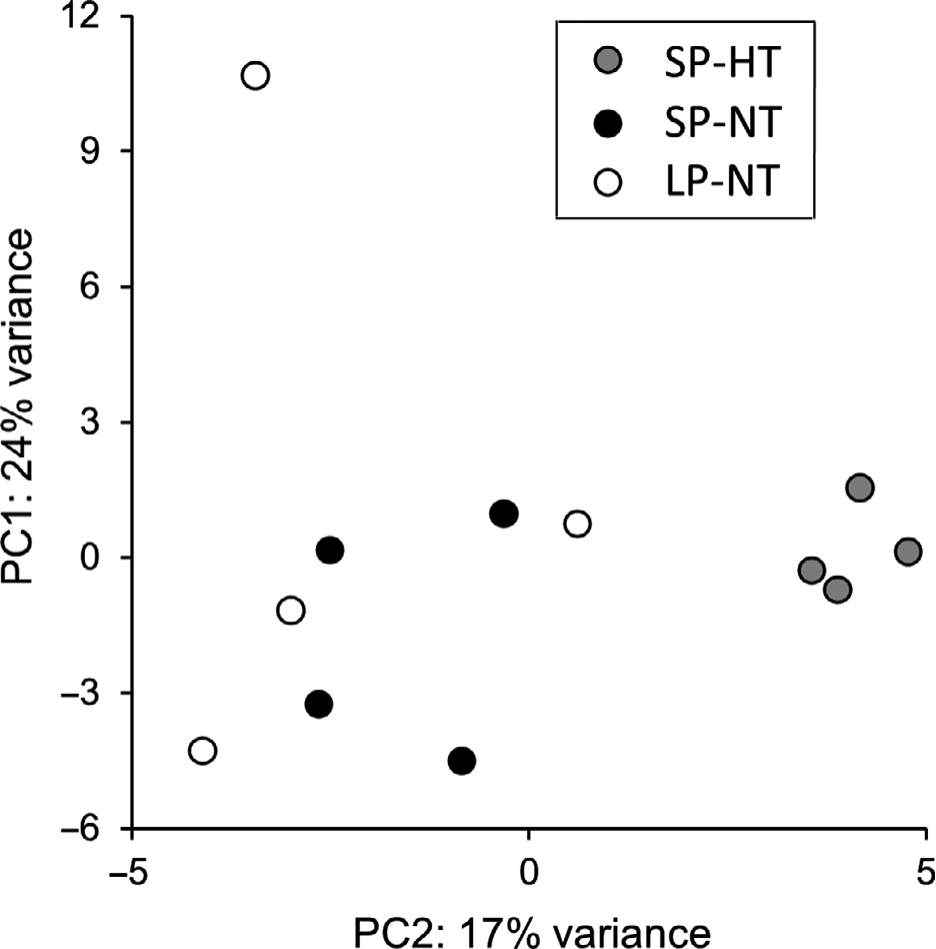
Principal component analysis. Differences in gene expression profiles of samples and sampling groups in the first two dimensions. The closer two dots, the more similar the gene expression profiles of two samples.

**Fig. 3:**
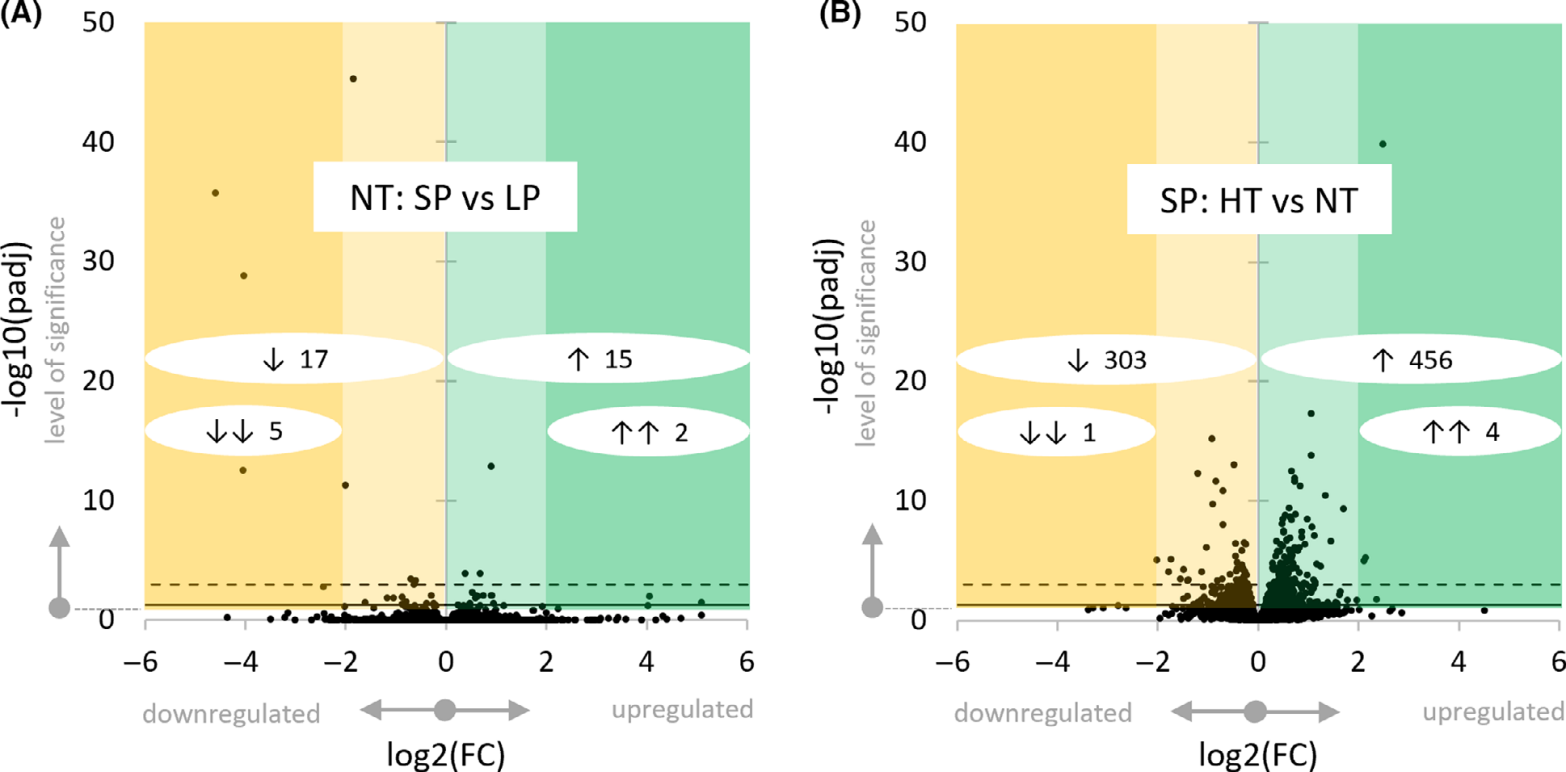
Volcano plots. Differential gene expression for the NT: SP vs LP comparison (A) and the SP: HT vs NT comparison (B). All mapped genes (dots) are represented by fold change (x‐axis) in dependence on significance (y‐axis). Differentially expressed genes are shown in the colored areas above the horizontal significance threshold (black line, −log10(0.05) = 1.3). Genes indicated with padj < 0.001 throughout this study are shown above the dotted line (−log10(0.001) = 3.0). The numbers of regulated genes per area are indicated (white circles expanding over the fold change values covered, with ↓↓ extremely downregulated in dark yellow area, ↓ downregulated in yellow area, ↑ upregulated in green area, ↑↑ extremely upregulated in dark green area).

Several analyses were used in this study to pin down the most relevant data. The principal component analysis (Fig. 2) visualizes the distance between each sample of this study by their respective overall gene expression. The volcano plots for each pairwise group comparison (Fig. 3) visualize each genes’ fold change dependent on its significance. In this study, differentially expressed genes were defined as statistically significant with a padj < 0.05 and any fold change. Highly statistically significant differentially expressed genes were stated as padj < 0.001.

### GO enrichment analysis

A GO enrichment analysis for each pairwise group comparison was performed to identify biological processes that are under regulation. The differentially expressed genes with padj < 0.05 were tested. All present genes with any padj‐value served as background to create the baseline of expectable regulation of biological processes. Data were analyzed using the PANTHER Overrepresentation Test (Released 20210224) based on the GO Ontology database (https://doi.org/10.5281/zenodo.4495804 Released 20210201), with Fisher’s exact as test type and false discovery rate correction. (PANTHER‐tool via http://geneontology.org/) [36, 37, 38]. The genes’ fold change was irrelevant for this test.

### Most extremely regulated genes

In addition to the threshold of significance, padj < 0.05, a threshold for the fold change, |log2(FC)| > 2, was applied to detect the most extremely regulated genes in the pairwise group comparisons.

### Indicator genes

As reviewed earlier, several hypothalamic systems including the circadian clock system, thyroid system, growth axis, and energy metabolism are of relevance in photoperiodic acclimation and/or orchestration of torpor [12]. Here, we specifically screened for differential expression of genes relevant to these systems in each pairwise group comparison. Immediate early genes of the Fos/Jun family were screened as representatives of transcriptional activity [39, 40, 41]. Our list of preselected indicator genes was extended to additional gene products and their receptors according to their annotation of biological function in the systems of interest according to AmiGO 2 (http://amigo.geneontology.org/amigo/search/annotation, 17.09.2021) and search in the UniProt knowledge base (https://www.uniprot.org/), again using *Mus musculus* as reference. The 68 preselected indicator genes are introduced in Table 1.

**Table 1:** The 68 preselected indicator genes. Genes of several key systems with potential regulatory function in torpor expression and photoperiodic status were screened for differential gene expression.

System	#	Gene	Gene product
Transcription	1 + 2	*Fos* + *Fosb*	Proto‐oncogene c‐fos + protein fosb
	3 + 4 + 5	*Jun* + *Junb* + *Jund*	Transcription factor ap‐1 / c‐jun + jun‐b + jun‐d
	6 + 7	*c‐Jun1* + *c‐Jun3*	c‐jun‐amino‐terminal kinase‐interacting protein 1 + 3
Clock	8	*Avp*	Vasopressin‐neurophysin 2‐copeptin
	9	*Avpr1a*	Vasopressin v1a receptor
	10 + 11	*Bmal1* + *Bmal2*	Brain and muscle arnt‐like 1 + 2
	12	*Bhlhe40*	Class e basic helix‐loop‐helix protein 40
	13	*Clock*	Circadian locomoter output cycles protein kaput
	14 + 15	*Cry1* + *Cry2*	Cryptochrome 1 + 2
	16	*Gpr50*	Melatonin‐related receptor
	17	*Id2*	DNA‐binding protein inhibitor id‐2
	18	*Mta1*	Metastasis‐associated protein mta1
	19 + 20	*Mtnr1a* + *Mtnr1b*	Melatonin receptor type 1a + 1b
	21 + 22 + 23	*Per1* + *Per2* + *Per3*	Period circadian protein homolog 1 + 2 + 3
	24	*Pml*	Protein pml
	25	*Ppp1cc*	Serine/threonine–protein phosphatase
	26	*Timeless*	Protein timeless homolog
	27	*Vip*	Vasoactive intestinal peptide
Thyroid	28 + 29	*Dio1* + *Dio2*	Iodothyronine deiodinase type i + ii
	30	*Dio3*	Thyroxine 5‐deiodinase
	31	*Mct8*	Monocarboxylate transporter 8
	32 + 33	*Thra* + *Thrb*	Thyroid hormone receptor alpha + beta
	34	*Trh*	Pro‐thyrotropin‐releasing hormone
	35 + 36	*Trhr* + *Trhr2*	Thyrotropin‐releasing hormone receptor 1 + 2
	37	*Tshr*	Thyrotropin receptor
	38	*Txnip*	Thioredoxin‐interacting protein
Growth	39	*GH*	Somatotropin / growth hormone
	40	*Sst*	Somatostatin
	41 ‐ 45	*Sstr1 ‐ Sstr5*	Somatostatin receptor type 1 ‐ 5
Metabolism	46 + 47	*Tas1r3* + *Tas1r2*	Taste receptor type 1 member 3 + 2
	48	*Glut1*	Glucose transporter member 1
	49 ‐ 52	*Glut3 ‐ 6*	Glucose transporter member 3 ‐ 6
	53	*P2ry1*	P2y purinoceptor 1
	54	*Fgfr1*	Fibroblast growth factor receptor 1
	55	*Insr*	Insulin receptor
	56	*Lepr*	Leptin receptor
	57	*Agrp*	Agouti‐related protein
	58	*Cartpt*	Cocaine‐ and amphetamine‐regulated transcript protein
	59	*Pomc*	Pro‐opiomelanocortin
	60 + 61	*Mc3r* + *Mc4r*	Melanocortin receptor 3 + 4
	62	*Npy*	Pro‐neuropeptide y
	63 + 64	*Npy1r* + *Npy2r*	Neuropeptide y receptor type 1 + 2
	65	*Qrfp*	Orexigenic neuropeptide qrfp
	66	*Qrfpr*	Pyroglutamylated rf‐amide peptide receptor
	67	*Ncam1*	Neural cell adhesion molecule 1
	68	*Vim*	Vimentin, cytosceleton of glial cells

## Results

To validate known mechanisms of photoperiodic acclimation and to generate hypotheses of underlying mechanisms of spontaneous torpor control, this study analyzed hypothalamic mRNA‐Seq data gained from LP‐acclimated normothermic, SP‐acclimated normothermic, and SP‐acclimated hypothermic Djungarian hamsters.

### Gene expression profiles

The *de novo* transcriptome assembly with 392,605,160 read pairs (Table S2) gained from 12 hypothalamic samples had on average 32,717,097 read pairs per sample. 171,923 transcripts of 868,657 transcripts in total were successfully mapped against more than 16.000 annotated genes of the reference *Mus musculus*.

A principal component analysis was conducted to describe the distance of each samples’ overall gene expression profile (Fig. 2). Samples were tightly clustered within the hypothermic SP‐acclimated group, less clustered within the normothermic SP‐acclimated group, and most diverse within the normothermic LP‐acclimated group. In the first dimension, the samples of the normothermic SP‐acclimated group scattered less than the samples of the normothermic LP‐acclimated group. In the second dimension, the gene expression profiles of the hypothermic group differed from the two normothermic groups, regardless of the photoperiodic acclimation.

The three sampling groups were used in two pairwise group comparisons: NT: SP vs LP to describe transcriptomic differences driven by photoperiod and SP: HT vs NT to unravel torpor‐driven differential gene expression. In the photoperiodic comparison (NT: SP vs LP), 32 of 16,199 genes (0.2%) reached the level of significance, whereas 759 of 16,195 genes, (4.7%) were significantly regulated during torpor (SP: HT vs NT).

The graphical representation of differential gene expression revealed few regulated genes with high fold changes between photoperiods (NT: SP vs LP; Fig. 3A) and many regulated genes with a mainly moderate fold change during torpor (SP: HT vs NT; Fig. 3B). While 15 genes were upregulated and 17 genes downregulated between photoperiods, the data show a torpor‐driven upregulation of 456 and downregulation of 303 genes within the hypothalamus.

### GO enrichment analysis

The photoperiodic comparison (NT: SP vs LP) revealed no enriched biological processes. During torpor (SP: HT vs NT), transcriptional, biosynthetic, cellular, and metabolic processes were enriched (Table 2). Listed are GO terms of those biological processes found to be regulated by less or more genes with padj < 0.05 than expected using all present genes as background.

**Table 2:** GO enrichment analysis. Data basis is the SP: HT vs NT comparison. Regulated genes with padj < 0.05 were tested, while all present genes were used as background. Listed are GO terms of those biological processes found to be regulated by less or more genes than expected using a false discovery rate (FDR) < 0.05.

Go biological process complete	Reference		Test				
	GO term	Hits		Expected	Fold enrich	Raw *P*‐value	FDR
Total		15604	748	‐	‐	‐	‐
Regulation of gene expression	GO:0010468	3855	258	185	1.4	6.42E‐09	3.30E‐05
Regulation of nucleic acid‐templated transcription	GO:1903506	2698	192	129	1.5	2.50E‐08	4.80E‐05
Regulation of transcription dna‐templated	GO:0006355	2694	192	129	1.5	1.87E‐08	4.12E‐05
Regulation of transcription by rna polymerase ii	GO:0006357	2054	140	98	1.4	2.87E‐05	2.46E‐02
Regulation of biosynthetic process	GO:0009889	3364	223	161	1.4	2.90E‐07	3.44E‐04
Regulation of rna biosynthetic process	GO:2001141	2701	192	129	1.5	2.53E‐08	4.33E‐05
Regulation of macromolecule biosynthetic process	GO:0010556	3149	218	151	1.4	1.40E‐08	4.31E‐05
Regulation of cellular biosynthetic process	GO:0031326	3293	220	158	1.4	1.85E‐07	2.59E‐04
Regulation of cellular macromolecule biosynthetic process	GO:2000112	3115	216	149	1.5	1.60E‐08	4.12E‐05
Regulation of cellular process	GO:0050794	8801	486	422	1.2	3.25E‐06	3.13E‐03
Regulation of cellular metabolic process	GO:0031323	4981	304	239	1.3	9.95E‐07	1.02E‐03
Cellular macromolecule metabolic process	GO:0044260	3655	225	175	1.3	4.98E‐05	4.04E‐02
Negative regulation of macromolecule metabolic process	GO:0010605	2333	156	112	1.4	2.33E‐05	2.11E‐02
Regulation of macromolecule metabolic process	GO:0060255	5077	320	243	1.3	1.14E‐08	4.37E‐05
Regulation of metabolic process	GO:0019222	5534	336	265	1.3	2.41E‐07	3.09E‐04
Regulation of nitrogen compound metabolic process	GO:0051171	4615	290	221	1.3	1.59E‐07	2.44E‐04
Regulation of nucleobase‐containing compound metabolic process	GO:0019219	3168	222	152	1.5	2.93E‐09	2.26E‐05
Regulation of primary metabolic process	GO:0080090	4781	297	229	1.3	3.20E‐07	3.52E‐04
Regulation of rna metabolic process	GO:0051252	2936	211	141	1.5	1.33E‐09	2.05E‐05

### Most extremely regulated genes

Within the predefined thresholds padj < 0.05 and |log2(FC)| > 2.0 (Table 3), seven genes were regulated by photoperiod (NT: SP vs LP) and five by torpor (SP: HT vs NT). In SP‐normothermia compared to LP‐normothermia (NT: SP vs LP), thyroxine 5‐deiodinase (*Dio3*) and chondroitin sulfate proteoglycan 4B (*Cspg4b*) were upregulated, while five genes were downregulated, namely lecithin retinol acyltransferase (*Lrat*), anaphase‐promoting complex subunit CDC26 (*Cdc26*), pro‐FMRFamide‐related neuropeptide VF (*Npvf*), thyrotropin subunit beta (*Tshb*), and glycoprotein hormones alpha chain (*Cga*). Four genes were upregulated in SP‐hypothermia compared with SP‐normothermia (SP: HT vs NT), namely RNA‐binding protein 3‐like (*Rbm3‐ps*), grainyhead‐like protein 2 homolog (*Grhl2*), adipolin (*C1qtnf12*), and short coiled‐coil protein‐like (*Gm16500*), while egl nine homolog 1 (*Egln1*) was downregulated.

**Table 3:** Most extremely regulated genes. All genes with padj < 0.05 and |log2(FC)| > 2.0 for both pairwise group comparisons are listed. A negative log2(FC) indicates downregulation, a positive log2(FC) upregulation relative to the respective baseline (LP‐NT for photoperiodic comparison and SP‐NT for torpor comparison).

	padj	log2(FC)	Gene	Gene product
NT: SP vs LP	0.038	5.1	*Dio3*	Thyroxine 5‐deiodinase
	0.010	4.1	*Cspg4b*	Chondroitin sulfate proteoglycan 4B
	< 0.001	−2.0	*Lrat*	Lecithin retinol acyltransferase
	0.002	−2.4	*Cdc26*	Anaphase‐promoting complex subunit CDC26
	< 0.001	−4.0	*Npvf*	Pro‐fmrfamide‐related neuropeptide VF
	< 0.001	−4.0	*Tshb*	Thyrotropin subunit beta
	< 0.001	−4.6	*Cga*	Glycoprotein hormones alpha chain
SP: HT vs NT	< 0.001	2.5	*Rbm3‐ps*	RNA‐binding protein 3‐like
	0.018	2.4	*Grhl2*	Grainyhead‐like protein 2 homolog
	< 0.001	2.1	*C1qtnf12*	Adipolin
	< 0.001	2.1	*Gm16500*	Short coiled‐coil protein‐like
	< 0.001	−2.0	*Egln1*	Egl nine homolog 1

### Indicator genes

Among the indicator genes, thyroxine 5‐deiodinase (*Dio3*) and period circadian protein homolog (*Per3*) were upregulated in SP‐normothermia compared with LP‐normothermia (NT: SP vs LP), while pro‐opiomelanocortin (*Pomc*) and melatonin‐related receptor (*Gpr50*) were downregulated. With a −log10(padj) of 45.3, *Gpr50* had the highest significance among all other regulated genes of this study. In SP‐hypothermia compared with SP‐normothermia (SP: HT vs NT), somatostatin receptor type 1 (*Sstr1*), glucose transporter member 3 (*Slc2a3 / Glut3*), and protein PML *(Pml)* were upregulated, whereas transcription factor AP‐1 *(Jun),* c‐Jun‐amino‐terminal kinase‐interacting protein 1 (*Mapk8ip1 / c‐Jun1*), class E basic helix‐loop‐helix protein 40 *(Bhlhe40),* and serine/threonine–protein phosphatase PP1‐gamma catalytic subunit *(Ppp1cc)* were downregulated.

Some indicator genes were not present in the hypothalamic mRNA‐Seq data of the Djungarian hamster, namely melatonin receptor type 1B (*Mtnr1b*), and taste receptor type 1 member 2 *(Tas1r2)*. Somatotropin / growth hormone *(GH)* had outlying values in NT: SP vs LP and was not present in SP: HT vs NT. All 68 predefined indicator genes are listed (Table 1). The significantly regulated indicator genes are presented (Table 4). The results of all indicator genes are provided in the supplementary material (Table S3).

**Table 4:** Regulated indicator genes. Indicator genes with padj < 0.05 for each pairwise group comparison. The results of all 68 preselected indicator genes, as introduced in Table 1, are provided in Table S3. A negative log2(FC) indicates downregulation, a positive log2(FC) upregulation relative to the respective baseline (LP‐NT for photoperiodic comparison and SP‐NT for torpor comparison).

	padj	log2(FC)	Gene	Gene product
NT: SP vs LP	0.038	5.1	*Dio3*	Thyroxine 5‐deiodinase
	< 0.001	0.7	*Per3*	Period circadian protein homolog 3
	0.015	−1.0	*Pomc*	Pro‐opiomelanocortin
	< 0.001	−1.8	*Gpr50*	Melatonin‐related receptor
SP: HT vs NT	0.013	0.3	*Sstr1*	Somatostatin receptor type 1
	0.043	0.2	*Glut3*	Glucose transporter member 3
	0.008	0.5	*Pml*	Protein PML
	< 0.001	−0.4	*Jun*	Transcription factor AP‐1 / c‐Jun
	0.041	−0.2	*c‐Jun1*	c‐Jun‐amino‐terminal kinase‐interacting protein 1
	< 0.001	−0.5	*Bhlhe40*	Class E basic helix‐loop‐helix protein 40
	0.048	−0.1	*Ppp1cc*	Serine/threonine–protein phosphatase

## Discussion

Seasonal acclimatizations of Djungarian hamsters including the use of spontaneous torpor have been intensely studied. Key hypothalamic mechanisms of long‐term acclimatizations in body mass and reproduction have been described. However, the proximate hypothalamic control of torpor episodes has remained rather unclear. Here, we analyzed hypothalamic transcriptomes of LP‐ and SP‐acclimated normothermic as well as SP‐acclimated torpid Djungarian hamsters to confirm existing knowledge from a photoperiodic context. We were assuming that the applied methods would also pin down relevant genes involved in torpor control.

Our approaches could well validate existing knowledge of photoperiodic gene expression changes in the hypothalamus, using a combination of indicator genes and those with most extreme regulation. Enrichment analyses failed to reveal enriched pathways due to short photoperiod exposure, suggesting that few very distinct changes maintain the photoperiodic response. During torpor however, many biological processes were enriched. This is likely reflecting mechanisms of the brain to compensate for the severely suppressed metabolism and decreased body temperature to maintain functional integrity. These supposedly adaptive mechanisms were also reflected in the most extremely regulated genes. Within the more hypothesis‐driven analysis of indicator genes, we found few differentially expressed genes with regulatory potential. Hence, although our applied methods are well able to detect gene expression changes relevant to regulate photoperiodic acclimation, distinct regulatory mechanisms might be overridden by vital overall adaptive mechanisms during torpor and are thus more difficult to pin down.

### Effects of the body temperature difference between sampling groups

Overall, our analyses revealed a substantially lower number of genes regulated by photoperiod (32) than by torpor (759). There is evidence that the difference between the terminal body temperatures of two sampling groups affects the number of regulated genes.

In the present study, 32 genes were regulated by photoperiod using SP‐acclimated hamsters which had expressed at least one torpor bout after sixteen weeks of acclimation (NT: SP vs LP). A previous study by Bao et al. also found 32 genes regulated by photoperiod using hamsters after eight weeks of acclimation to a 10:14 light:darkness SP‐light regime [18], hence probably prior to torpor manifestation. In thirteen‐lined ground squirrels that are obligate and seasonal hibernators with deep, multi‐day torpor bouts, far more pronounced seasonal differences in gene expression were described (1206 genes in the summer vs. interbout normothermia comparison). These differences might result from species‐specific differences in control mechanisms that might be more pronounced and hardwired in an obligate seasonal hibernator as opposed to a photoperiodic daily heterotherm [42].

Our present study showed 759 genes to be regulated during torpor at ZT04 (456 up, 303 down, proportion 6 : 4) with a body temperature difference of 10 °C (SP: HT vs NT). A previous study by Cubuk et al. [17] revealed 284 regulated genes during torpor entrance at ZT01 (181 up, 103 down, proportion 6 : 4) with a body temperature difference of about 6 °C [17]. It is reasonable to assume that the severe depression of metabolic rate, resulting in low body temperatures and thus high body temperature differences between the torpid and the nontorpid group (Fig. 1), leads to more severe gene expression changes as opposed to purely photoperiodic changes in metabolically active and normothermic animals (Fig. 2, Fig. 3). In thirteen‐lined ground squirrels, comparable relationships between body temperature and number of differentially expressed genes were found during the hibernation season. Within the same seasonal state, only few hypothalamic genes were regulated between sampling groups with a small body temperature difference, whereas many genes were regulated between sampling groups with a large body temperature difference [42].

### Direction of regulation and transcriptional activity

Especially in small endotherms, a metabolic downstate comes along with low body temperatures. Probable side effects are a slowed down transcription as well as post‐transcriptional effects, such as prolongation of mRNA half‐life or altered translation [43, 44]. However, the proportion between up‐ and downregulated genes is high. In Djungarian hamster, the proportion is 6 : 4 in both torpor entry compared with SP‐normothermia [17] and deep torpor compared with SP‐normothermia (Fig. 3). In thirteen‐lined ground squirrel, the proportion is 6 : 4 in torpor entrance vs. interbout normothermia and 7 : 3 in torpor arousal vs. interbout normothermia [42]. These proportions indicate that metabolic downstates are rather actively regulated in the hypothalamus than simply caused by slowed down transcription in the torpid sampling group.

However, the immediate early genes *c‐Jun1* and *Jun* were downregulated during torpor, which is consistent with hypothalamic gene expression data from Djungarian hamster and hibernating golden‐mantled ground squirrel [39, 45]. No members of the Fos family were regulated in this study. Although a member of the Jun family dimerizes with a member of the Fos family to become transcriptionally active, a downregulation of one family might already suggest an overall transcriptional downregulation. Yet, this interpretation might be too simple, given the lack of anatomical precision in a whole hypothalamus approach, which cannot differentiate nucleus‐specific activation or deactivation of immediate early genes. Furthermore, mRNA of transcriptional factors has reasonably a shorter half‐life than mRNA of genes related to metabolism and structure [46].

### GO enrichment analyses

The GO enrichment analysis did not identify enriched processes in the photoperiodic comparison (NT: SP vs LP), but revealed enriched transcriptional, biosynthetic, cellular, and metabolic processes during torpor (SP: HT vs NT; Table 2). These findings indicate that photoperiodic acclimations are rather driven by very distinct mechanisms that are not picked up by this general method in a whole hypothalamus transcriptome approach. During torpor however, severe remodeling of many major processes comes to the fore, likely reflecting general adaptive and protective mechanisms of the brain to the metabolic depression and low body temperature. These dominant changes are likely to override distinct control signals. This picture might be underlined by the fact that the small number of genes regulated by photoperiod showed high fold changes, whereas most of the large number of genes regulated in torpor showed moderate fold changes (Fig. 3). Furthermore, highly specialized, essential genes probably not investigated in detail are annotated to few or even no biological processes. In the GO enrichment analysis, they are methodologically suppressed by genes involved in general and therefore well‐studied functions, which are annotated to various biological processes. Besides this, the GO enrichment analysis does not respect a regulated genes’ biological relevance in terms of fold change and padj‐values.

### Most extremely regulated genes

In this study, most extremely regulated genes were defined as genes with padj < 0.05 and |log2(FC)] > 2.0. These strict parameters were chosen because they were able to pick up the known key players of physiologically relevant gene expression changes in the photoperiodic comparison. Applying the same thresholds in the torpor comparison, we identified genes that appear to rather reflect adaptive mechanisms of the brain.

#### Comparison SP‐NT vs. LP‐NT

Comparison between photoperiods revealed seven most extremely regulated genes. In SP, *Dio3* was among the upregulated genes. *Dio3* is a T3 catabolic enzyme, converting the bioactive hormone into inactive or less active derivates. Upregulation in SP is consistent with previous data, demonstrating that *Dio3*‐dependent reduction of hypothalamic T3 availability is a crucial driver of SP‐acclimation in Djungarian hamsters [47, 48, 49, 50, 51].

*Tshb,* encoding for the thyrotropin beta subunit, was downregulated in normothermic SP‐acclimated hamsters. Downregulation of *Tshb* in *Pars tuberalis* has previously been identified as crucial interface of melatonin signaling and hypothalamic gene expression in many species [52, 53, 54]. In birds and mammals, thyrotropin interconnects the circadian clock with the thyroid system and indirectly controls reproduction and the molt circle [55, 56, 57, 58].

*Cga* had, with a log2(FC) of −4.6, the most extreme (down)regulation during SP‐acclimation (Fig. 3A). *Cga* contributes as glycoprotein hormones alpha chain with the hormone‐specific beta subunits TSHB, LHB, and FSHB to the formation of the hypothalamic hormones thyrotropin, lutropin, and follitropin, respectively [59].

*Lrat*, the lecithin retinol acetyltransferase, was downregulated in SP‐acclimated hamsters. This enzyme is important for vision. It does not only play a role in the retina, but also play a role in other tissues to metabolize vitamin A [60, 61]. In the photoperiodic context, retinoic acid‐signaling genes were reported to be differentially regulated within the hypothalamus and may play a role in body mass regulation and energy metabolism [62, 63].

*Npvf* was downregulated in SP. *Npvf* encodes RFamide‐related peptides (*Rfrp*), such as kisspeptin and *Qrfp*. Kisspeptin plays a role in the photoperiodic control of reproduction, while *Qrfp* is relevant for hypometabolism in mice [5, 64, 65]. *Npvf* was previously found to be downregulated in hypothalamus during early SP acclimation (SP08) [18].

The other most extremely regulated genes were not directly linked to photoperiodic acclimation. The uncharacterized protein LOC408066, also known as chondroitin sulfate proteoglycan 4B at locus *Cspg4b* and upregulated in SP‐acclimated hamsters, is related to diverse topics in several fields. As structural macromolecules of connective tissues’ extracellular matrix, *Cspg4b* might be related to brain plasticity. *Cdc26*, downregulated in SP‐acclimated hamsters, has been studied in Chinese hamster ovary cells only, but might contribute as part of the anaphase‐promoting complex to brain structure and metabolism [66].

#### Comparison SP‐HT vs. SP‐NT

Using the same thresholds, we picked up five regulated genes during torpor that appear to reflect adaptive mechanisms. During torpor, the cold‐shock protein *Rbm3* was upregulated. Studies on natural and enforced hypothermia in the hibernating 13‐line ground squirrels and in cooled mice suggest that *Rbm3* and other cold‐inducible RNA‐binding proteins within the brain might enable RNA stability and protein synthesis at low body temperatures, which is important for synaptic regeneration and therefore structural plasticity [67, 68, 69].

*Egln1* was downregulated during torpor. Although its biological relevance is still unclear, it has been linked to ischemia and hypoxia in mouse and human due to, for example, its suppression of hypoxia‐inducible factor 1α (HIF‐1α) [70, 71, 72]. The importance of avoiding blood clotting and tolerating low oxygen content in the context of torpor and hibernation has been previously discussed [73, 74].

The genes upregulated during torpor have not been in focus of torpor research so far. Adipolin has been studied regarding diabetes, obesity, and cardiovascular diseases such as ischemia. It increases insulin sensibility and favors glucose tolerance and uptake, which might play a role for energy gain and storage in the torpid hypothalamus [75]. *Grhl2* appears to play a role in hearing loss. The expression of *Grhl2* and HIF‐1α was linked [76]. At this point, no information is available on the function of *Gm16500*, also known as predicted gene 16500 or short coiled‐coil protein‐like.

Overall, the most regulated genes during torpor also reflect major structural changes, rather than distinct signaling mechanisms. This was already concluded from our previous study, which analyzed gene expression during torpor entrance, although time point of sampling, bioinformatic approach, reference organism, and consequently the actual regulated genes were different [17].

### Indicator genes

In a more targeted approach to identify mechanisms involved in torpor control, we specifically screened a number of indicator genes from potentially relevant hypothalamic systems (Table 1) as reviewed earlier [12]. These genes were chosen based on existing knowledge from physiological data regarding single gene products, their receptors, or annotation to a particular system of interest such as the circadian clock system, thyroid system, growth axis, and energy metabolism. Only few of the 68 indicator genes were found significantly regulated in either group comparison. It is possible that only single components of a given system are altered and thereby change their output. Moreover, relevant genes in the brain often occur in a nucleus‐specific way and expression changes might disappear when sequencing the whole hypothalamus. Furthermore, each gene product has naturally several functions in diverse systems, whereas current knowledge is incomplete.

#### Comparison SP‐NT vs. LP‐NT

Four of 68 indicator genes were differentially regulated by photoperiod (Table 4). Among those genes, we found *Dio3* to be upregulated with an extremely high log2(FC) of 5.1 (see paragraph ‘Most extremely regulated genes’). By contrast, *Per3* was upregulated with high significance in SP‐normothermia. *Per3* is a photoinducible clock gene oscillating with circadian rhythm [77, 78]. Since we only assessed gene expression data at one circadian time point (ZT04), we are unable to draw conclusions about the clockwork here. Differential gene expression might reflect photoperiod‐specific clockwork changes. *Pomc* was downregulated in SP‐acclimated hamsters’ hypothalamus, which is consistent with earlier studies on the hamsters’ energy homeostasis. *Pomc* might be involved in controlling the photoperiodic body mass acclimation of Djungarian hamsters [79, 80].

The gene with the most extreme significance of this study was *Gpr50*, downregulated in SP‐acclimated hamsters. This photoperiodic downregulation of *Gpr50* has previously been demonstrated [50, 51, 81]. Besides its role in photoperiodic acclimations, studies on mice indicate that lack of *Gpr50* augments fasting‐induced torpor and suppresses leptin responsiveness as well as thyrotropin‐release hormone [82]. Thus, a photoperiodically reduced expression of *Gpr50* might be a torpor‐permissive factor, although the gene is not regulated during individual torpor bouts. Furthermore, *Gpr50* seems to play a major role in overall energy metabolism, as knockout mice had a higher food intake, a higher locomotor activity, and higher basal metabolic rate, yet a lower body mass and fat mass [83]. *Gpr50* is likely to be located on the X‐chromosome. Although irrelevant for this NT: SP vs LP comparison with two males and two females in each group, the location of a gene on sex chromosomes might play a role for the interpretation of the results of the other comparison. In SP: HT vs NT, three males and one male sampled in torpor were used to compare their gene expression with those of two males and two females sampled on a torpor‐free day.

#### Comparison SP‐HT vs. SP‐NT

Seven of 68 indicator genes were differentially expressed during torpor (Table 4). The somatostatin receptor *Sstr1* was upregulated. It has previously been demonstrated that the GH axis is regulated by photoperiod and involved in torpor control [84]. Downregulation of the axis by a somatostatin receptor 1 agonist has been demonstrated to reinforce torpor behavior. Upregulation of GH axis components during torpor in our current data might either result from the pulsatile nature of this system or represent highly dynamic regulation over the different torpor phases. The GH axis should be investigated during torpor in more detail, since this system seems to play a major role not only in the Djungarian hamster but also in hibernators as the European hamster [85].

*Glut3*, the most important glucose and galactose transporter in the brain, was upregulated during torpor. This might be related to an enhanced hexose transport to the brain. Plasma glucose levels are reduced during torpor, while hypoglycemia seems to be rather a consequence than a requisite for torpor [86, 87]. Either more glucose transporters ensure sufficient glucose availability to the brain despite low glucose levels, or the low plasma glucose levels are caused by an enhanced glucose consumption of the brain during torpor and in preparation for torpor arousal, mediated by more glucose transporters. Both mechanisms might help to maintain functionality of the brain during torpor, which is an energy‐saving mechanism integrated in the overall short photoperiod‐adjusted energy balance, yet energy‐consuming during certain stages of torpor orchestration [88]. Besides possible roles of the upregulation of a glucose transporter in the hypothalamus, the known photoperiodic changes in the glucose metabolism of tanycytes were reviewed elsewhere [89]. Furthermore, *Glut3* expression can be mediated by HIF‐1α [90, 91], which again links glucose metabolism with ischemia and hypoxia.

Although uninvestigated in the Djungarian hamster so far*, Pml* is annotated to the mouse’ circadian clock. A loss of *Pml* causes a downregulation of *Per2, Per1, Cry1, Bmal1,* and *Npas2* expression in the mouse’ suprachiasmatic nucleus [92]. Its upregulation in torpor is hard to interpret because of a hardly regulated clock on hypothalamic resolution and various other functions of *Pml*, like in the Tgf‐beta signaling pathway [93].

Besides a downregulation of the immediate early genes *Jun* and *c‐Jun1* (see paragraph ‘Direction of regulation and transcriptional activity’), *Bhlhe40* and *Ppp1cc* were downregulated in torpor as well, both with proven functions in the mouse’ circadian clock with unclear consequences. *Bhlhe40* regulates the phase of several clock genes, perhaps in a novel feedback loop different from the positive regulator *CLOCK/BMAL* and the negative regulator *PER/CRY* [94, 95]. *Ppp1cc* is an enzyme with countless substrates, which plays a role in as much biological processes including energy metabolism of cells [96] and regulation of time period [97].

Some indicator genes were annotated in the reference *Mus musculus* but were absent in this study’s data on hypothalamic mRNA expressed by the Djungarian hamster (Table S3). This might either result from sequence divergence between the two species in combination with the application of a similarity threshold, or from absence of the gene in the hypothalamus of *Phodopus sungorus*. Phylogenetic relations within the Rodentia, genetic distances between species, and heterozygosity between subpopulations are under constant research, facing genetic bottlenecks, and inbreed of laboratory animals [98, 99, 100].

The lack of functional *Mtnr1b* in the Djungarian hamster has been previously described, underlining the functionality of our data approach [101]. To our knowledge, the absence of the sweet taste receptor *Tas1r2* in its hypothalamus has not been described so far. Interestingly, *Tas1r2* null mice do have more glucose nonresponsive tanycytes, while a respectable number of glucosensitive tanycytes use other, hitherto unknown, mechanisms for glucose‐sensing [102]. The Djungarian hamster can compensate the lack of *Tas1r2* with *Tas1r3* and other mechanisms, since it is able to time not only spontaneous torpor to photophase, but also fasting‐induced torpor to a known feeding schedule [103].

## Conclusion

Taken together, our approach was able to validate existing knowledge about hypothalamic key players of photoperiodic control. For example, analysis of both, most extremely regulated and predefined indicator genes, confirmed a downregulation of *Dio3* expression during short photoperiod adaptation of Djungarian hamsters. Less distinct results were found when applying the same parameters to pin down torpor control mechanisms. Considering both, most extremely regulated and indicator genes, only the regulation of *Egln1* and *Glut3* stood out immediately, since both genes and gene products can be related by the transcriptional complex HIF. Interestingly, the short‐term factor torpor led to a substantially higher number of significantly regulated genes compared with the long‐term factor season, whereby the majority of torpor‐related changes could be attributed to remodeling processes, likely reflecting general adaptive and protective mechanisms of the brain to the metabolic depression and low body temperature. The enrichment of these transcriptional, biosynthetic, cellular, and metabolic processes emphasizes that metabolic downstates are actively regulated and not simply caused by slowed down transcription. The torpor‐related gene expression analysis produced less distinct results but has generated valuable data to generate new hypotheses about proximate hypothalamic regulation of individual torpor episodes. More targeted approaches allowing nucleus or even cell‐specific analyses of mechanisms might help to further narrow down respective physiological mechanisms. This study provides a basis for future research in biomolecular and neuroendocrine studies on spontaneous torpor. It contributes a valuable, open‐source data set for future screening of further genes of interest.

## Conflict of interest

The authors declare that the research was conducted in the absence of any commercial or financial relationships that could be construed as a potential conflict of interest.

## Author contributions

All authors conceived the project. EH performed the animal work. EH and VD performed surgery and sacrifice. JB developed the bioinformatical pipeline. EH performed the bioinformatics, statistics, and analyses under supervision of JB. EH, VD, and AH interpreted the data. EH wrote the manuscript, which was carefully revised by all authors. All authors agree to be accountable for the content of the work.

## Supporting information


**Doc S1**. Comparative transcriptomics of the djungarian hamster hypothalamus.
**Doc S2**. Processing pipeline ‐ executed commands from trim‐galore to rsem.
**Doc S3**. R script with R session info ‐ executed normalization (from *P*‐value to padj), statistics (pairwise group comparison), principal component analysis, heatmap, setup of the r‐software and installed packages.
**Fig. S1**. Core body temperature patterns of SP‐acclimated hamsters.
**Table S1**. Background information on hamsters.
**Table S2**. Quality control of RNA‐Seq data and accession numbers of data depositories.

## Data Availability

The data that support the findings of this study are openly available in NCBI's Gene Expression Omnibus and are accessible through GEO Series accession number GSE179663 (https://www.ncbi.nlm.nih.gov/geo/query/acc.cgi?acc=GSE179663). An overview of accession numbers within SRA and GEO is attached as Table S2. The data processing pipeline from raw Illumina data to non‐normalized gene expression is attached as Doc S2. Both ruby‐scripts were uploaded to SourceForge (https://sourceforge.net/projects/prepare‐transcript‐to‐gene‐map/). The R‐script with R‐session info is attached as Doc S3.
