# Supplementary material for: Comparative transcriptomics of the Djungarian hamster hypothalamus during short photoperiod acclimation and spontaneous torpor
Source: FEBS Open Bio. 2021 Dec 20;12(2):443–59. doi: 10.1002/2211-5463.13350 (PMC8804604; doi:10.1002/2211-5463.13350)
Supplement: Supplementary file 1 — Doc S1. Comparative transcriptomics of the djungarian hamster hypothalamus. Doc S2. Processing pipeline ‐ executed commands from trim‐galore to rsem. Doc S3. R script with R session info ‐ executed normalization (from P‐value to padj), statistics (pairwise group comparison), principal component analysis, heatmap, setup of the r‐software and installed packages. Fig. S1. Core body temperature patterns of SP‐acclimated hamsters. Table S1. Background information on hamsters. Table S2. Quality control of RNA‐Seq data and accession numbers of data depositories. [file FEB4-12-443-s001.docx]

**Supplementary File 1**

Haugg E, Borner J, Diedrich V, Herwig A (2021)

Comparative Transcriptomics of the Djungarian Hamster Hypothalamus

during Short Photoperiod Acclimation and Spontaneous Torpor

Correspondence to: elena.haugg@uni-ulm.de

This file includes:

Supplementary Table 1: Background information on hamsters.

Supplementary Figure 1: Core body temperature patterns of SP-acclimated hamsters.

Supplementary Table 2: Quality control of RNA-Seq data and accession numbers

of data depositories.

Supplementary Table 3: Results of the 68 predefined indicator genes.

**Supplementary Table 1: Background information on hamsters (n = 12).** Hamster identification number, gender (f for female, m for male), body mass, fur index, age, and duration of short photoperiod (SP) exposure at several key dates. Body mass and fur index are usual parameters to assess the acclimation state. The initial body mass was measured at the beginning of the experiment, when eight of twelve hamsters were transferred to SP. Until termination, hamsters in SP reduced body mass, while hamsters in LP retained or increased their body mass, yet with a high variation ^[[1]](#footnote-1)^. The fur index was assessed from 1 (brown light summer fur) to 6 (white isolating winter fur) according to Figala et al. 1973 ^[[2]](#footnote-2)^. All SP-acclimated hamsters were capable of torpor, while their torpor incidence, which is the number of torpor bouts per days of the observation interval, varied ^1^. The terminal core body temperature (Tb) is indicated as plotted in **Supplementary Figure 1.** Several parameters were only assessed for hamsters which were transferred to SP, since four hamsters remained without radiotelemetry transmitter in long photoperiod (LP).

| **hamster ID** | **gender** | **body mass** | | | **fur index** | **age [weeks] at** | | |
| --- | --- | --- | --- | --- | --- | --- | --- | --- |
|  |  | **initial [g]** | **sacrifice [g]** | **difference [%]** | **sacrifice** | **SP** | **surgery** | **sacrifice** |
| EH03-26 | m | 41.9 | 28.7 | -31.5 | 5.0 | 18.3 | 29.4 | 32.1 |
| EH03-33 | m | 31.3 | 25.4 | -18.8 | 3.0 | 15.6 | 27.4 | 29.6 |
| EH03-10 | m | 42.3 | 26.4 | -37.6 | 3.5 | 19.4 | 29.7 | 37.3 |
| EH03-02 | f | 29.4 | 26.3 | -10.5 | 5.5 | 22.4 | 33.6 | 40.4 |
| EH03-36 | f | 28.3 | 25.8 | -8.8 | 4.5 | 15.6 | 27.7 | 29.9 |
| EH03-38 | m | 41.2 | 29.5 | -28.4 | 3.5 | 13.6 | 25.9 | 28.0 |
| EH03-37 | f | 25.4 | 25.5 | 0.4 | 3.0 | 15.6 | 27.9 | 29.9 |
| EH03-19 | m | 37.1 | 25.8 | -30.5 | 3.0 | 15.7 | 29.6 | 34.1 |
| EH03-LP1 | m | 38.2 | 37.1 | -2.9 | 1.0 | - | - | 32.0 |
| EH03-LP2 | f | 30.0 | 32.5 | 8.3 | 1.0 | - | - | 30.0 |
| EH03-LP3 | m | 32.2 | 36.9 | 14.6 | 1.0 | - | - | 29.9 |
| EH03-LP5 | f | 29.6 | 29.0 | -2.0 | 1.0 | - | - | 29.7 |

| **hamster ID** | **duration [weeks] in SP at** | | **observation** | | **torpor incidence** | | **sampling group** | **terminal Tb [°C]** |
| --- | --- | --- | --- | --- | --- | --- | --- | --- |
|  | **surgery** | **sacrifice** | **interval [weeks]** | **torpor bouts [#]** | **observation interval** | **last 7 full days** |  |  |
| EH03-26 | 11.1 | 13.9 | 2.7 | 12 | 0.6 | 0.9 | SPHT | 23.6 |
| EH03-33 | 11.9 | 14.0 | 2.1 | 4 | 0.3 | 0.4 | SPHT | 24.0 |
| EH03-10 | 10.3 | 17.9 | 7.6 | 30 | 0.6 | 0.7 | SPHT | 25.0 |
| EH03-02 | 11.1 | 18.0 | 6.9 | 14 | 0.3 | 0.4 | SPHT | 25.9 |
| EH03-36 | 12.1 | 14.3 | 2.1 | 2 | 0.1 | 0.3 | SPNT | 34.9 |
| EH03-38 | 12.3 | 14.4 | 2.1 | 6 | 0.4 | 0.3 | SPNT | 35.3 |
| EH03-37 | 12.3 | 14.3 | 2.0 | 1 | 0.1 | 0.0 | SPNT | 35.4 |
| EH03-19 | 13.9 | 18.4 | 4.6 | 10 | 0.3 | 0.7 | SPNT | 35.5 |
| EH03-LP1 | - | - | - | - | - | - | LPNT | - |
| EH03-LP2 | - | - | - | - | - | - | LPNT | - |
| EH03-LP3 | - | - | - | - | - | - | LPNT | - |
| EH03-LP5 | - | - | - | - | - | - | LPNT | - |

**Supplementary Figure 1: Core body temperature patterns of SP-acclimated hamsters (n = 8).** The graph shows the day of sacrifice and the day before. Time is given in *Zeitgeber* time (ZT), with lights on at ZT0 and lights off at ZT08. Photophases are indicated (grey bars on the x-axis). After at least one observed torpor bout per hamster (see also **Supplementary Table 1**), sacrifice was carried out approximately ZT04 (vertical line), either in deep torpor (HT, n = 4, black lines) or without torpor expression (NT; n = 4, grey lines).


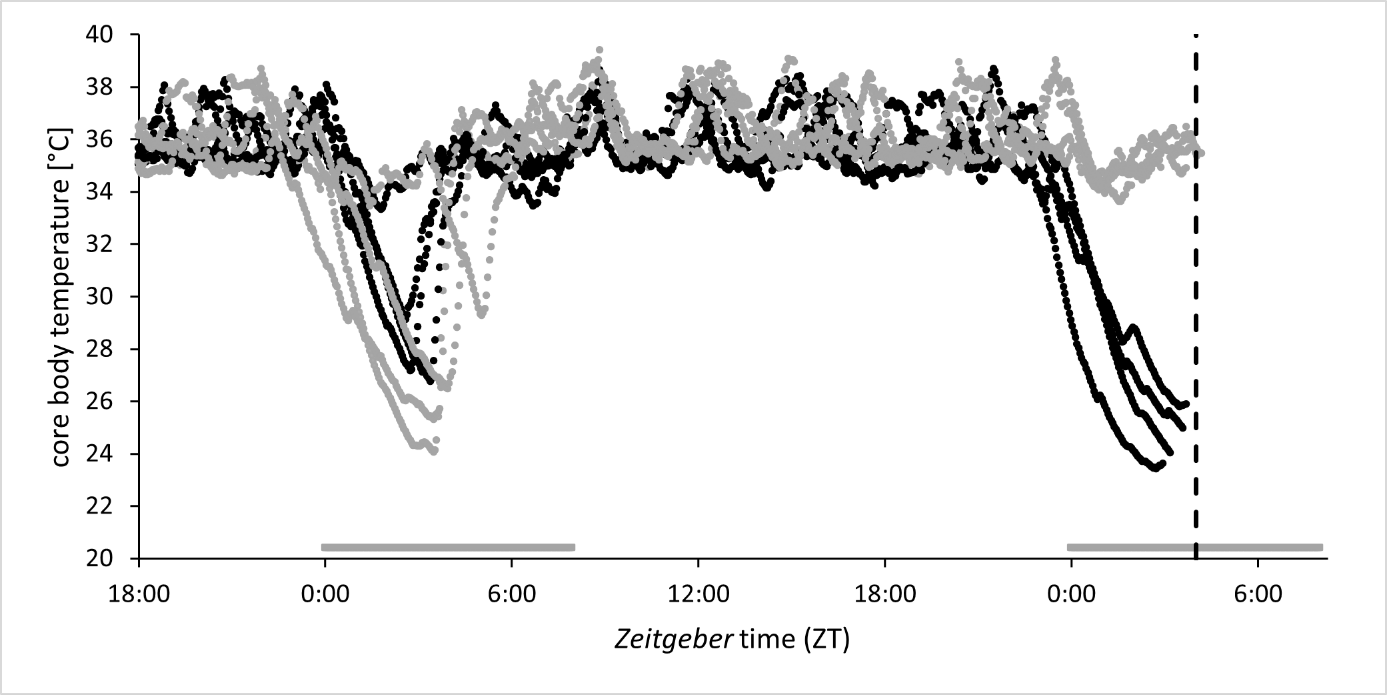


**Supplementary Table 2: Quality control of RNA-Seq data and accession numbers of data depositories.** Quality control was performed with FastQC ^[[3]](#footnote-3)^. Sequence length was throughout 151 base pairs. The GC-content was 48 or 49 % per file, per sample and in total. The number of total read pairs ranged from 30,375,548 to 35,052,887 with a mean of 32,717,097 per sample. In total, 392,605,160 read pairs were included in the original *de novo* assembly before mapping against the reference was performed. Each sample is indicated by the photoperiod (SP or LP), metabolic state at termination (HT or NT), and a running number within the cohort EH03. All data discussed in this study are accessible as indicated through GEO Series accession number GSE179663 (<https://www.ncbi.nlm.nih.gov/geo/query/acc.cgi?acc=GSE179663>).

| **read pairs** | **sample ID** | **BioSample in BioProject PRJNA743775** | **raw illumina data in SRA Study SRP326941** | **non-normalized gene expression in GEO Series GSE179663** |
| --- | --- | --- | --- | --- |
| 33,069,666 | SPHT02 | SAMN20060157 | SRR15041647 | GSM5425878 |
| 30,498,292 | SPHT10 | SAMN20060158 | SRR15041646 | GSM5425879 |
| 31,130,806 | SPHT26 | SAMN20060159 | SRR15041645 | GSM5425880 |
| 30,375,548 | SPHT33 | SAMN20060160 | SRR15041644 | GSM5425881 |
| 34,994,187 | SPNT19 | SAMN20060161 | SRR15041643 | GSM5425882 |
| 35,006,001 | SPNT36 | SAMN20060162 | SRR15041642 | GSM5425883 |
| 34,055,106 | SPNT37 | SAMN20060163 | SRR15041651 | GSM5425884 |
| 35,052,887 | SPNT38 | SAMN20060164 | SRR15041650 | GSM5425885 |
| 32,690,972 | LPNT01 | SAMN20060153 | SRR15041653 | GSM5425874 |
| 32,951,578 | LPNT02 | SAMN20060154 | SRR15041652 | GSM5425875 |
| 31,571,032 | LPNT03 | SAMN20060155 | SRR15041649 | GSM5425876 |
| 31,209,085 | LPNT05 | SAMN20060156 | SRR15041648 | GSM5425877 |

**Supplementary Table 3: Results of the 68 predefined indicator genes.** (Table see next page). Results for both pairwise group comparisons, namely NT: SP vs LP and SP: HT vs NT. Indicator genes with padj < 0.05 (black fields with white labelling) were presented in the main document **(Table 4)**. A negative log2(FC) indicates downregulation (yellow), a positive log2(FC) upregulation (green) relative to the according baseline (LP-NT for photoperiodic comparison, SP-NT for torpor comparison). “Not present” (grey) marks genes which were not present in one or both pairwise group comparisons but annotated in the reference *Mus musculus*. “Outlier” (grey) replaces a padj-value given the outlying count according to the statistics performed with DESeq2 ^[[4]](#footnote-4)^.

**Supplementary Table 3: Results of the 68 predefined indicator genes.** (Legend text see previous page).

|  | **#** | **NT: SP vs LP** | | | **SP: HT vs NT** | | | **gene** | **gene product** |
| --- | --- | --- | --- | --- | --- | --- | --- | --- | --- |
|  |  | **padj** | **-log10 (padj)** | **log2 (FC)** | **padj** | **-log10 (padj)** | **log2 (FC)** |  |  |
| **transcription** | 1 | 0.991 | 0.0 | -1.02 | 0.975 | 0.0 | -0.02 | *Fos* | proto-oncogene c-Fos |
|  | 2 | 0.992 | 0.0 | -0.12 | 0.973 | 0.0 | 0.04 | *Fosb* | protein fosB |
|  | 3 | 0.991 | 0.0 | -0.13 | 0.000 | 5.4 | -0.44 | *Jun* | transcription factor AP-1 / c-Jun |
|  | 4 | 0.991 | 0.0 | -0.25 | 0.817 | 0.1 | 0.07 | *Junb* | transcription factor jun-B |
|  | 5 | 0.991 | 0.0 | -0.03 | 0.926 | 0.0 | -0.02 | *Jund* | transcription factor jun-D |
|  | 6 | 0.996 | 0.0 | -0.01 | 0.041 | 1.4 | -0.16 | *c-Jun1* | c-Jun-amino-terminal kinase-interacting protein 1 |
|  | 7 | 0.991 | 0.0 | 0.04 | 0.052 | 1.3 | -0.14 | *c-Jun3* | c-Jun-amino-terminal kinase-interacting protein 3 |
| **clock** | 8 | 0.996 | 0.0 | -0.03 | 0.322 | 0.5 | 0.60 | *Avp* | vasopressin-neurophysin 2-copeptin |
|  | 9 | 0.967 | 0.0 | -0.40 | 0.913 | 0.0 | -0.07 | *Avpr1a* | vasopressin V1a receptor |
|  | 10 | 0.073 | 1.1 | -0.22 | 0.330 | 0.5 | -0.16 | *Bmal1* | brain and muscle ARNT-like 1 |
|  | 11 | 0.997 | 0.0 | 0.00 | 0.768 | 0.1 | 0.21 | *Bmal2* | brain and muscle ARNT-like 2 |
|  | 12 | 0.991 | 0.0 | 0.11 | 0.000 | 13.0 | -0.47 | *Bhlhe40* | class E basic helix-loop-helix protein 40 |
|  | 13 | 0.991 | 0.0 | 0.08 | 0.973 | 0.0 | 0.01 | *Clock* | circadian locomoter output cycles protein kaput |
|  | 14 | 0.991 | 0.0 | 0.08 | 0.519 | 0.3 | -0.10 | *Cry1* | cryptochrome-1 |
|  | 15 | 0.991 | 0.0 | 0.16 | 0.670 | 0.2 | -0.09 | *Cry2* | cryptochrome-2 |
|  | 16 | 0.000 | 45.3 | -1.85 | 0.721 | 0.1 | -0.20 | *Gpr50* | melatonin-related receptor |
|  | 17 | 0.837 | 0.1 | -0.21 | 0.051 | 1.3 | -0.38 | *Id2* | DNA-binding protein inhibitor ID-2 |
|  | 18 | 0.995 | 0.0 | -0.02 | 0.607 | 0.2 | 0.09 | *Mta1* | metastasis-associated protein MTA1 |
|  | 19 | 0.991 | 0.0 | -1.18 | outlier | | | *Mtnr1a* | melatonin receptor type 1A |
|  | 20 | not present | | | not present | | | *Mtnr1b* | melatonin receptor type 1B |
|  | 21 | 0.991 | 0.0 | 0.25 | 0.379 | 0.4 | -0.24 | *Per1* | period circadian protein homolog 1 |
|  | 22 | 0.089 | 1.1 | 0.36 | 0.228 | 0.6 | 0.24 | *Per2* | period circadian protein homolog 2 |
|  | 23 | 0.000 | 3.9 | 0.68 | 0.814 | 0.1 | 0.08 | *Per3* | period circadian protein homolog 3 |
|  | 24 | 0.996 | 0.0 | -0.02 | 0.008 | 2.1 | 0.55 | *Pml* | protein PML |
|  | 25 | 0.996 | 0.0 | -0.02 | 0.048 | 1.3 | -0.14 | *Ppp1cc* | serine/threonine-protein phosphatase |
|  | 26 | 0.991 | 0.0 | 0.19 | 0.871 | 0.1 | 0.14 | *Timeless* | protein timeless homolog |
|  | 27 | 0.991 | 0.0 | -0.28 | 0.353 | 0.5 | 0.21 | *Vip* | vasoactive intestinal peptide |
| **thyroid** | 28 | 0.991 | 0.0 | 0.90 | 0.433 | 0.4 | 0.73 | *Dio1* | iodothyronine deiodinase type I |
|  | 29 | 0.952 | 0.0 | -0.37 | 0.801 | 0.1 | 0.11 | *Dio2* | iodothyronine deiodinase type II |
|  | 30 | 0.038 | 1.4 | 5.10 | outlier | | | *Dio3* | thyroxine 5-deiodinase |
|  | 31 | 0.992 | 0.0 | 0.03 | 0.918 | 0.0 | -0.04 | *Mct8* | monocarboxylate transporter 8 |
|  | 32 | 0.996 | 0.0 | 0.01 | 0.775 | 0.1 | -0.04 | *Thra* | thyroid hormone receptor alpha |
|  | 33 | 0.991 | 0.0 | 0.17 | 0.550 | 0.3 | 0.15 | *Thrb* | thyroid hormone receptor beta |
|  | 34 | 0.991 | 0.0 | 0.10 | 0.614 | 0.2 | 0.10 | *Trh* | pro-thyrotropin-releasing hormone |
|  | 35 | 0.991 | 0.0 | 0.14 | 0.449 | 0.3 | 0.21 | *Trhr* | thyrotropin-releasing hormone receptor |
|  | 36 | 0.991 | 0.0 | 0.20 | 0.845 | 0.1 | 0.09 | *Trhr2* | thyrotropin releasing hormone receptor 2 |
|  | 37 | 0.991 | 0.0 | -0.10 | 0.868 | 0.1 | 0.08 | *Tshr* | thyrotropin receptor |
|  | 38 | 0.991 | 0.0 | -0.13 | 0.143 | 0.8 | -0.25 | *Txnip* | thioredoxin-interacting protein |
| **growth** | 39 | outlier | | | not present | | | *GH* | somatotropin / growth hormone |
|  | 40 | 0.991 | 0.0 | 0.10 | 0.829 | 0.1 | 0.05 | *Sst* | somatostatin |
|  | 41 | 0.632 | 0.2 | -0.25 | 0.013 | 1.9 | 0.33 | *Sstr1* | somatostatin receptor type 1 |
|  | 42 | 0.991 | 0.0 | -0.08 | 0.066 | 1.2 | 0.28 | *Sstr2* | somatostatin receptor type 2 |
|  | 43 | 0.997 | 0.0 | 0.00 | 0.560 | 0.3 | 0.16 | *Sstr3* | somatostatin receptor type 3 |
|  | 44 | 0.991 | 0.0 | 0.25 | 0.855 | 0.1 | 0.11 | *Sstr4* | somatostatin receptor type 4 |
|  | 45 | 0.991 | 0.0 | -0.22 | 0.920 | 0.0 | 0.09 | *Sstr5* | somatostatin receptor type 5 |
| **metabolism** | 46 | 0.991 | 0.0 | -0.05 | 0.805 | 0.1 | -0.08 | *Tas1r3* | taste receptor type 1 member 3 |
|  | 47 | not present | | | not present | | | *Tas1r2* | taste receptor type 1 member 2 |
|  | 48 | 0.997 | 0.0 | 0.01 | 0.304 | 0.5 | -0.16 | *Glut1* | glucose transporter member 1 |
|  | 49 | 0.991 | 0.0 | -0.08 | 0.043 | 1.4 | 0.23 | *Glut3* | glucose transporter member 3 |
|  | 50 | 0.991 | 0.0 | 0.24 | 0.790 | 0.1 | -0.19 | *Glut4* | glucose transporter member 4 |
|  | 51 | 0.995 | 0.0 | 0.04 | 0.623 | 0.2 | 0.25 | *Glut5* | glucose transporter member 5 |
|  | 52 | 0.991 | 0.0 | 0.13 | 0.838 | 0.1 | -0.06 | *Glut6* | glucose transporter member 6 |
|  | 53 | 0.991 | 0.0 | 0.30 | 0.418 | 0.4 | 0.24 | *P2ry1* | P2Y purinoceptor 1 |
|  | 54 | 0.997 | 0.0 | 0.00 | 0.836 | 0.1 | -0.03 | *Fgfr1* | fibroblast growth factor receptor 1 |
|  | 55 | 0.991 | 0.0 | 0.08 | 0.803 | 0.1 | -0.04 | *Insr* | insulin receptor |
|  | 56 | 0.991 | 0.0 | 0.10 | 0.448 | 0.3 | 0.23 | *Lepr* | leptin receptor |
|  | 57 | 0.996 | 0.0 | -0.06 | outlier | | | *Agrp* | agouti-related protein |
|  | 58 | 0.991 | 0.0 | -0.10 | 0.398 | 0.4 | 0.28 | *Cartpt* | cocaine- and amphetamine-regulated transcript protein |
|  | 59 | 0.015 | 1.8 | -1.05 | 0.183 | 0.7 | 0.40 | *Pomc* | pro-opiomelanocortin |
|  | 60 | 0.991 | 0.0 | -0.21 | 0.829 | 0.1 | 0.11 | *Mc3r* | melanocortin receptor 3 |
|  | 61 | 0.869 | 0.1 | -0.57 | 0.491 | 0.3 | 0.40 | *Mc4r* | melanocortin receptor 4 |
|  | 62 | 0.063 | 1.2 | -0.62 | 0.061 | 1.2 | -0.54 | *Npy* | pro-neuropeptide Y |
|  | 63 | 0.991 | 0.0 | -0.14 | 0.063 | 1.2 | 0.27 | *Npy1r* | neuropeptide Y receptor type 1 |
|  | 64 | 0.991 | 0.0 | -0.23 | 0.442 | 0.4 | 0.36 | *Npy2r* | neuropeptide Y receptor type 2 |
|  | 65 | 0.996 | 0.0 | -0.11 | outlier | | | *Qrfp* | orexigenic neuropeptide QRFP |
|  | 66 | 0.991 | 0.0 | 0.46 | 0.479 | 0.3 | 0.47 | *Qrfpr* | pyroglutamylated RF-amide peptide receptor |
|  | 67 | 0.991 | 0.0 | 0.05 | 0.379 | 0.4 | -0.10 | *Ncam1* | neural cell adhesion molecule 1 |
|  | 68 | 0.991 | 0.0 | -0.33 | 0.431 | 0.4 | 0.27 | *Vim* | vimentin, cytosceleton of glial cells |

1. Haugg E, Herwig A, Diedrich V (2021) Body temperature and activity adaptation of short photoperiod exposed Djungarian hamsters (Phodopus sungorus): Timing, traits, and torpor. Front Physiol. https://doi.org/10.3389/fphys.2021.626779 [↑](#footnote-ref-1)
2. Figala J, Hoffmann K, Goldau G (1973) Zur Jahresperiodik beim Dsungarischen Zwerghamster Phodopus sungorus Pallas. Oecologia 12:89–118. https://doi.org/10.1007/BF00345511 [↑](#footnote-ref-2)
3. Andrews S (2010) FastQC: A Quality Control Tool for High Throughput Sequence Data. <https://www.bioinformatics.babraham.ac.uk/projects/fastqc/> [↑](#footnote-ref-3)
4. Love MI, Anders S, Huber W (2020) Analyzing RNA-seq data with DESeq2. <http://bioconductor.org/packages/release/bioc/vignettes/DESeq2/inst/doc/DESeq2.html#pvaluesNA> (20210622). [↑](#footnote-ref-4)
